# Reduced secreted clusterin as a mechanism for Alzheimer-associated *CLU* mutations

**DOI:** 10.1186/s13024-015-0024-9

**Published:** 2015-07-16

**Authors:** Karolien Bettens, Steven Vermeulen, Caroline Van Cauwenberghe, Bavo Heeman, Bob Asselbergh, Caroline Robberecht, Sebastiaan Engelborghs, Mathieu Vandenbulcke, Rik Vandenberghe, Peter Paul De Deyn, Marc Cruts, Christine Van Broeckhoven, Kristel Sleegers

**Affiliations:** VIB Department of Molecular Genetics, University of Antwerp – CDE, Building V Universiteitsplein 1, B-2610 Antwerpen, Belgium; Institute Born-Bunge, Laboratory of Neurochemistry and Behavior, University of Antwerp, Antwerp, Belgium; Department of Neurology and Memory Clinic, Hospital Network Antwerp Middelheim and Hoge Beuken, Antwerp, Belgium; Department of Psychiatry and Memory Clinic, University of Leuven and University Hospitals Leuven Gasthuisberg, Leuven, Belgium; Laboratory for Cognitive Neurology, Department of Neurology, University of Leuven and University Hospitals Leuven Gasthuisberg, Leuven, Belgium; Department of Neurology and Alzheimer Research Center, University of Groningen and University Medical Center Groningen, Groningen, The Netherlands

**Keywords:** Alzheimer’s disease, Clusterin, Mutations, Rare variant, β-chain, Cell secretion, Golgi

## Abstract

**Background:**

The clusterin (*CLU*) gene has been identified as an important risk locus for Alzheimer’s disease (AD). Although the actual risk–increasing polymorphisms at this locus remain to be identified, we previously observed an increased frequency of rare non-synonymous mutations and small insertion-deletions of *CLU* in AD patients, which specifically clustered in the β-chain domain of CLU. Nonetheless the pathogenic nature of these variants remained unclear.

Here we report a novel non-synonymous *CLU* mutation (p.I360N) in a Belgian Alzheimer patient and have explored the pathogenic nature of this and 10 additional *CLU* mutations on protein localization and secretion *in vitro* using immunocytochemistry, immunodetection and ELISAs.

**Results:**

Three patient-specific *CLU* mutations in the β-chain (p.I303NfsX13, p.R338W and p.I360N) caused an alteration of the subcellular CLU localization and diminished CLU transport through the secretory pathway, indicative of possible degradation mechanisms. For these mutations, significantly reduced CLU intensity was observed in the Golgi while almost all CLU protein was exclusively present in the endoplasmic reticulum. This was further confirmed by diminished CLU secretion in HEK293T and HEK293 FLp-In cell lines.

**Conclusions:**

Our data lend further support to the contribution of rare coding *CLU* mutations in the pathogenesis of neurodegenerative diseases. Functional analyses suggest reduced secretion of the CLU protein as the mode of action for three of the examined *CLU* mutations. One of those is a frameshift mutation leading to a loss of secreted protein, and the other two mutations are amino acid substitutions in the disulfide bridge region, possibly interfering with heterodimerization of the α- and β-chain of CLU.

**Electronic supplementary material:**

The online version of this article (doi:10.1186/s13024-015-0024-9) contains supplementary material, which is available to authorized users.

## Background

Genome-wide association studies have provided compelling evidence for a role of genetic variation in the clusterin gene (*CLU* aka *APOJ*) in susceptibility of Alzheimer’s disease (AD) [1, 2]. The strongest association was found for the common single nucleotide polymorphism (SNP) rs11136000 located in a non-coding, intronic *CLU* region. The causal variant explaining the association however remains to be identified.

We previously demonstrated that, independent of the reported association signal, rare (minor allele frequency < 1 %) non-synonymous and insertion/deletion mutations in the *CLU* β–chain are associated with increased AD risk [3]. Numerous of these rare mutations are predicted as pathogenic based on PolyPhen and SIFT, which are programs considering evolutionary conservation, sequence-based and structure-based features [4, 5]. Nonetheless, interpreting the functional consequences of *CLU* variants remains challenging given the plentitude of biological functions in e.g. protein chaperoning, apoptosis, and complement activation. Most interestingly, CLU is associated with AD-related pathways: as an Aβ chaperone CLU can modulate both clearance and aggregation of Aβ, CLU acts as a lipid transporter in brain and it is involved in neuronal apoptosis (reviewed in [6]). In brain, CLU is predominantly secreted by astrocytes [7, 8].

The clusterin protein is synthesized as a 60–80 kD precursor protein undergoing endoproteolysis resulting in α and β chains which are joined together by disulfide bonds. This glycosylated heterodimeric protein is constitutively secreted. This secreted isoform (sCLU) results from translation of the full clusterin mRNA that codes for the 449 residues-long protein and contains a 22-mer leader sequence. The sCLU is induced during stress and inflammation and is believed to have cytoprotective and chaperone functions [9]. It was shown that two differentially processed *CLU* isoforms in brain (of 501 and of 449 amino acids respectively) both produce secreted proteins [10]. In addition to sCLU forms, less abundant non-secreted, intracellular CLU – cytoplasmic and truncated nuclear – forms have been described. These result from alternative splicing events leading to shorter mRNA variants lacking exon 5 [11] or exon 2 [12]. Opposed to the protective effects of the extracellular sCLU forms, the intracellular forms (iCLU) are linked to cell cytotoxicity [13].

Interestingly the AD-associated non-synonymous mutations and insertion/deletions in *CLU* were mainly present in the β-chain domain, at the interface with the α-chain, presuming a role for the β protein subunit in disease [3]. We designed an assay for high-throughput analysis of the *CLU* coding regions based on multiplex amplification of specific targets for resequencing (MASTR™) and massive parallel sequencing. Here we report the identification of a novel mutation (p.I360N) in a Belgian Alzheimer patient. Moreover, we explored the effect of this mutation, together with 10 previously reported coding *CLU* mutations, on subcellular localization and secretion of CLU protein. We found that multiple *CLU* mutations led to reduced CLU secretion, as shown by the higher retention in the endoplasmic reticulum (ER) and concomitant decreased concentration in the Golgi apparatus. These findings illustrate that rare Alzheimer mutations in the *CLU* β-chain can deregulate normal CLU secretion and lead to protein degradation in ER before subsequent trafficking towards Golgi.

## Results

### Identification of a novel AD mutation in the β-chain domain

Mutation analysis of the *CLU* β-chain encoding exons in 74 Alzheimer patients identified a yet unreported (from 1000GP, EVS) missense mutation (p.I360N) in one patient with clinically probable late-onset AD (onset age of 78 years), and two missense mutations previously observed in patients and controls [3] (Table 1). The p.I360N variant lies within the central cysteine-rich region adjacent to Cys354, Cys357 and Cys365 forming disulfide bonds at the interface between the *CLU* β*-*chain and α-chain. Three *in silico* prediction programs suggested a damaging effect of this variant (Table 1).

**Table 1:** Rare non-synonymous *CLU* mutations identified in 74 AD patients

Gene location^a^	DNA^b^	Protein^c^	Protein^d^	dbSNP	This study	Previous study [3]	Protein location	PolyPhen2 (PSIC)	SIFT	PMUT	EVS (EA)	1000GP
Exon 6	c.1079 T > A	p.I360N	p.I308N	^−^	1 AD	-	β-chain	Possible (0.677)	Not tolerated (0.00)	Pathological (0.9659)	-	-
Exon 5	c.701G > A	p.R234H	p.R182H	^−^	1 AD	2 AD, 1 C	α-chain	Probably (0.995)	Tolerated (0.15)		0.0002	0.001
Exon 5	c.764C > T	p.T255I	p.T203I	rs4127629	1 AD	5 AD, 6 C	β-chain	Benign (0.041)	Tolerated (0.29)	Pathological (0.6284)	0.003	0.001

Abbreviations: *AD* Alzheimer patient, C control individual, AAO Age of Onset, y years

^a^Gene location position according to *CLU* transcript with 9 coding exons [NM_001831.2]

^b^Numbering according to CLU mRNA sequence starting at the A of the ATG translation initiation codon in [NM_001831.2]

^c^Numbering according to CLU protein sequence [NP_001822.2 consisting of 501 AA]

^d^For easy comparison to Exome Variant Server, numbering according to updated CLU protein sequence [NP_001822.3 consisting of 449 AA] is given. Mutations were compared to our findings previously described in Belgian patients [3]. Predictions of pathogenicity was performed using Polyphen2 (benign/possibly damaging/probably damaging), SIFT (tolerated/not tolerated) and PMUT (neutral/pathological). Minor allele frequency (MAF) was compared to Exome Variant Server (EVS) in European American individuals (EA) and 1000 GenomesProject (1000GP)

### Functional follow-up of small insertion/deletion and missense mutations in *CLU*

The p.I360N mutation and nine previously reported *CLU* variants [3] were selected for further functional investigation. This selection included disease-associated *CLU* mutations with predicted pathogenic effects, a predicted benign variant (p.A309T) and two variants observed in both patients and controls (p.P322L, p.N369H) (Table 2). Of note, p.A309T and T445_D447del occur as a double mutation in Belgian, but not in French or Canadian patients, hence these mutations were investigated individually as well as in combination. In total, 11 different constructs were generated.

**Table 2:** Rare coding β-chain variants transfected in cells

#	Gene location^a^	Protein^b^	Protein^c^	PolyPhen2 (PSIC)	SIFT
Alzheimer specific *CLU* mutations					
1	**Exon 5**	**p.I303NfsX13**	**p.I251NfsX13**	**-**	**-**
2	Exon 5	p.A309T	p.A257T	Benign (0.32)	Tolerated (0.65)
3	**Exon 6**	**p.R338W**	**p.R286W**	**Probable (1.00)**	**Not tolerated (0.00)**
4	**Exon 6**	**p.T345M**	**p.T293M**	**Probable (1.00)**	Tolerated (0.09)
5	**Exon 6**	**p.I360N**	**p.I308N**	**Possible (0.68)**	**Not tolerated (0.00)**
6	**Exon 7**	**p.E431Q**	**p.E379Q**	**Possible (0.83)**	Tolerated (0.35)
7	**Exon 7**	**p.T440M**	**p.T388M**	**Possible (0.92)**	**Not tolerated (0.01)**
8	**Exon 8**	**p.T445_D447del**	**p.T393_D395del**	**-**	**-**
*CLU* mutations observed in patients and controls					
9	Exon 5	p.P322L	p.P270L	Benign (0.03)	Tolerated (0.25)
10	**Exon 7**	**p.N369H**	**p.N317H**	**Probable (1.0)**	Tolerated (0.15)

^a^Gene location position according to *CLU* transcript with 9 coding exons [NM_001831.2]

^b^Numbering according to CLU protein sequence [NP_001822.2]

^c^For easy comparison to variants reported in Exome Variant Server (EVS), numbering according to recently updated CLU protein sequence [NP_001822.3 consisting of 449 AA] is given. The first 8 mutations were observed in AD patients only, variants 9 and 10 in patients and controls from this and previous study [3]

Belgian carriers of p.T445_D447del also harbor p.A309T. Predictions of pathogenicity of missense mutations was performed using Polyphen2 (benign/possibly damaging/probably damaging) and SIFT (tolerated/not tolerated). Small indels and non-synonymous mutations with predicted pathogenic effects are marked in bold

`

### The *CLU* β-chain variants affect CLU localization in HeLa cells

**Fig. 1:**
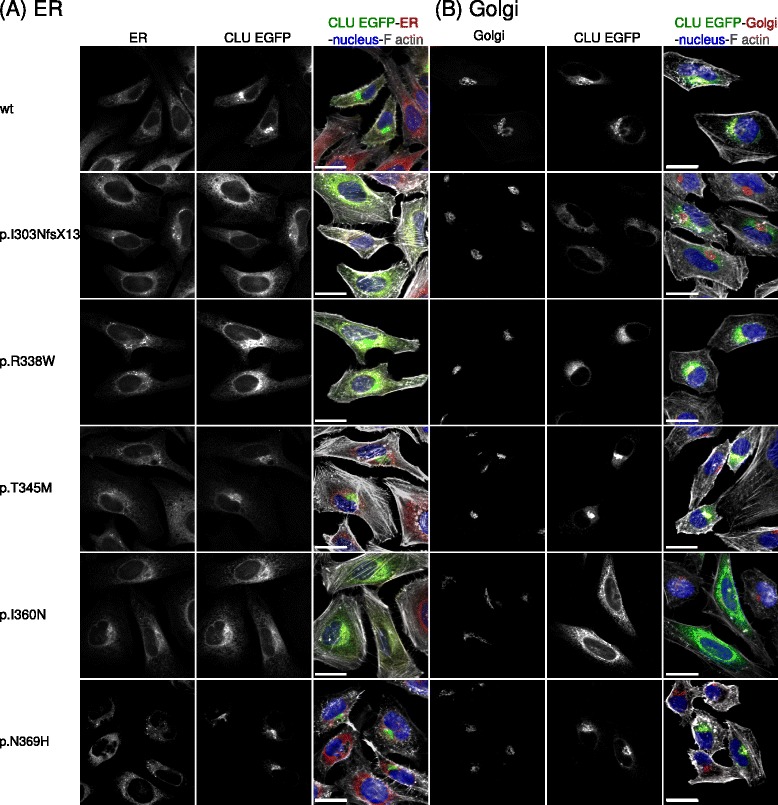
Localization of CLU-EGFP for different CLU coding mutations. Immunocytochemistry of CLU with markers for Golgi or ER was performed on HeLa cells transiently overexpressing wild-type CLU-EGFP or variants p.I303NfsX13, p.R338W, p.T345M, p.I360N and p.N369H. **a** The localization of CLU in ER is altered for p.I303NfsX13, p.R338W and p.I360N, compared to wt, p.T345M and p.N369H. **b** The CLU localization tends to concentrate in Golgi for wt and most missense mutations, (p.T345M and p.N369H are shown as example) while mutations p.I360N, p.I303NfsX13 and p.R338W showed decreased EGFP signal in the Golgi. Merged images for CLU (EGFP, green), ER (PDI, red) or Golgi (Giantin, red), nucleus (DAPI, blue), and actin cytoskeleton (Phalloidin, grey). The scale bar represents 25 μm

To investigate whether the AD-associated *CLU* mutations affect the subcellular localization of CLU, we performed live cell imaging and immunocytochemistry experiments on HeLa cells transiently transfected with EGFP-tagged wild-type (wt) and 11 mutated *CLU* constructs. Of all mutants, 3 (p.R338W, p.I303NfsX13 and p.I360N) showed altered CLU localization compared to wild-type CLU and other variants (Fig. 1).

**Fig. 2:**
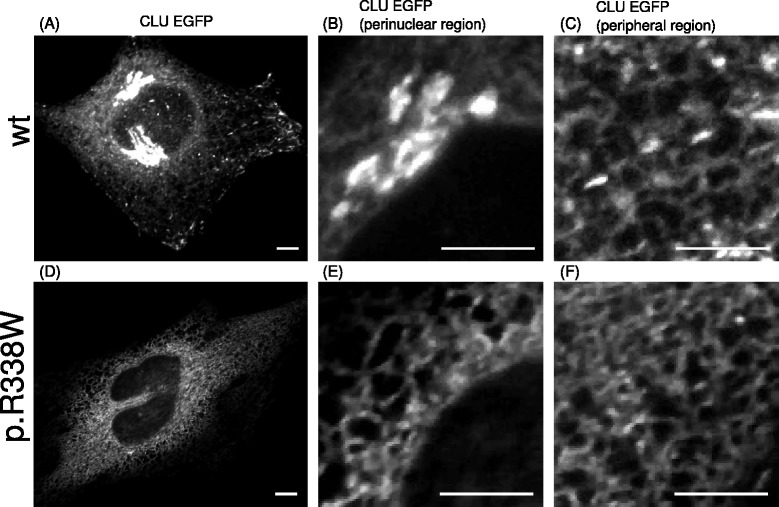
Localization of CLU-EGFP in HeLa cells. The distribution of *CLU*–EGFP wild-type (**a**-**c**) and one of the *CLU* coding mutations p.R338W (**d**-**f**) is shown for the entire cell (**a** and **d**), and for enlarged regions in the perinuclear region (**b** and **e**) and the cell periphery (**c** and **f**) of the same cell. For wild-type CLU the most intense EGFP signal was present in the Golgi (**a** and **b**) and in vesicles in the cytoplasm (**a** and **c**) in addition to the ER, whereas for p.R338W the CLU EGFP seemed almost exclusively present in the ER (**d**-**f**). No CLU-EGFP was present in the nucleus (**b** and **e**). The scale bar represents 5 μm

The CLU-EGFP signal of wild-type *CLU*-expressing cells was predominantly present in distinct zones in the cytoplasm close to the nucleus (Fig. 2a-b). This location represents the Golgi apparatus, as established by the typical cisterna-shaped structures (Fig. 2b) and the perfect overlap when co-staining with a Golgi marker (Giantin) (Fig. 1b). CLU-EGFP could also be detected at lower intensities in the endoplasmic reticulum (ER): ER membrane structures could be clearly discerned, which also colocalized with a marker for the ER (PDI) (Figs. 1a, 2c). In addition, more intense EGFP signals were detected in vesicular structures, reminiscent of endosomes (Fig. 2c). No nuclear clusterin staining was detected, consistent with the secreted CLU construct lacking the nuclear retention signal (NM_001831.1) (Fig. 2b).

When examining the different *CLU* mutations for their cellular distribution of CLU-EGFP, the high intensity signal accumulation in the Golgi was clearly lost for *CLU* mutations p.I303NfsX13, p.R338W and p.I360N, when compared to wild-type and all other *CLU* coding variants (Figs. 1 and 2d-f and Additional file 1: Figure S1, Additional file 2: Figure S2 and Additional file 3: Figure S3).

**Fig. 3:**
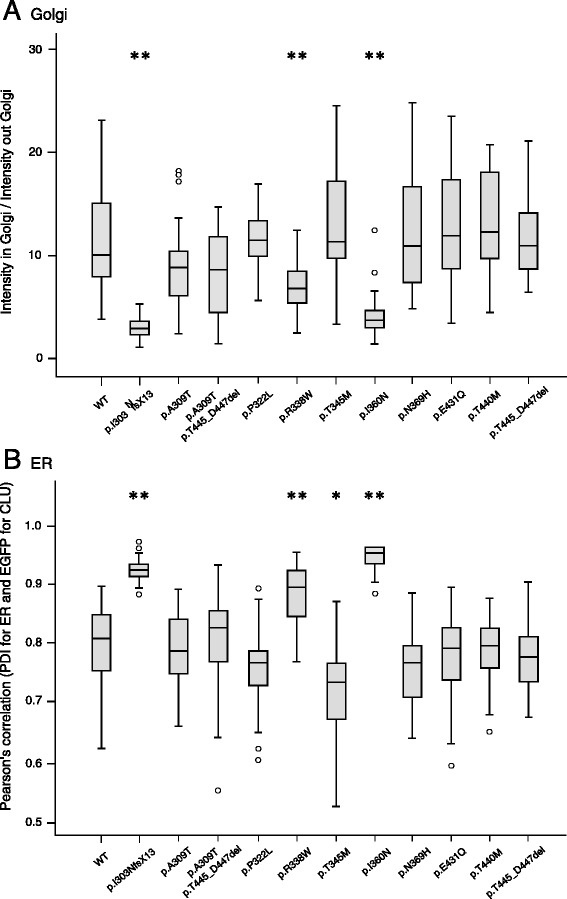
Quantifications of CLU-EGFP intensity in ER and Golgi of HeLa cells expressing CLU mutations. **a** For Golgi the ratio of the mean CLU-EGFP intensity inside versus outside the Golgi region was determined after excluding cells with low EGFP signal outside Golgi. **b** For ER, the Pearson’s correlation coefficient between the fluorescence channels of CLU-EGFP and PDI is represented. Measurements were performed on individual cells with ImageJ. Data are represented as median values, with lower and upper quartiles and the value ranges (whiskers) with a minimum of 20 cells per *CLU* variant. (*p < 0.05; **p < 0.01 after Bonferroni correction)

To quantify this, we measured the relative amount of CLU-EGFP signal in the Golgi region for the different cell lines and found significantly reduced CLU-EGFP intensity in Golgi for p.I303NfsX13, p.R338W and p.I360N (Fig. 3a). For these three mutants, CLU-EGFP signal was almost exclusively present in ER, which was verified by a drastic increase of the colocalization measurement of the CLU-EGFP signal with an ER marker (Fig. 3b).

For p.T345M, a decreased ER localization was observed, albeit no change was observed for this variant in Golgi. The altered ER signal is most likely due to the large intra-variant variability (Fig. 3b).

Most conclusive effects were observed for p.I303NfsX13, p.R338W and the novel mutation p.I360N. Mutant p.I303NfsX13 results in a premature stop codon and is predicted to be degraded by the cellular mRNA surveillance machinery prior to processing through the secretory pathway. This is confirmed by its increased ER levels and protein absence in Golgi (Fig. 3).

### The *CLU* β-chain variants alter CLU secretion in HEK293 cells

**Fig. 4:**
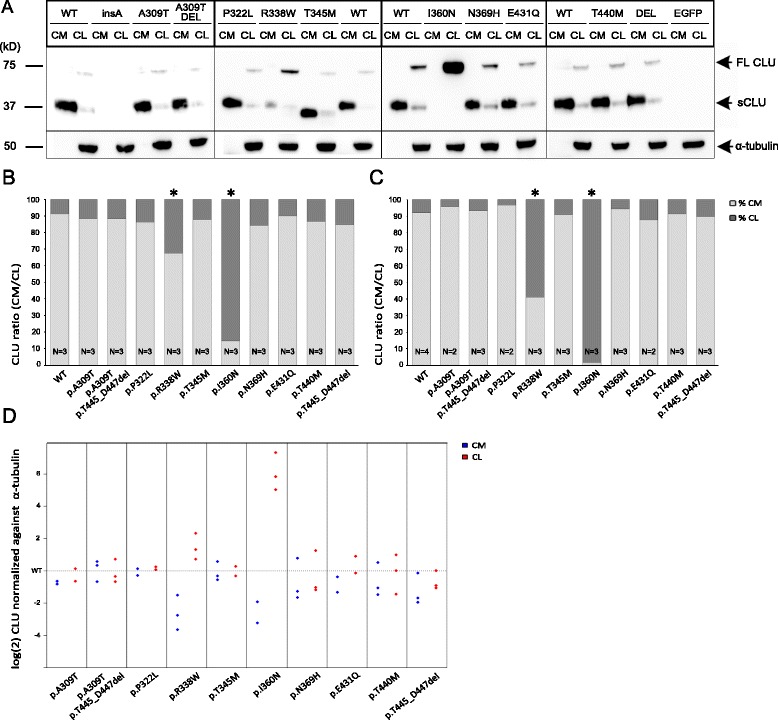
Visualization and quantification of CLU secretion for different *CLU* coding mutations in HEK Flp-In cells. **a** Immunoblot visualization of CLU Flp-In cells, showing lower sCLU levels in cell media (CM) and increased expression of full length (FL) CLU in cellular lysates (CL) for p.R338W and p.I360N. Alpha-tubulin staining is incorporated as an equal loading control. **b** ELISA quantification of CLU in HEK Flp-In CM and CL, presented as relative proportions of total CLU in CM and CL, displays a significant ratio decrease for p.R338W and p.I360N (*p < 1×10^−4^). N corresponds to the number of independent experiments. **c** Quantification of western blot band intensities in CM and CL of HEK Flp-In cells, presented as relative proportions of total CLU in CM and CL, with a significantly decreased CLU ratio of CM/CL for p.R338W and p.I360N (*p < 1×10^−4^). N corresponds to the number of independent experiments. **d** Western blot CLU intensities in CM (blue) and CL (red) normalized against α-tubulin and logarithmically rescaled against wt. **a**-**d** Mutant p.I303NfsX13 was not included in these experiments because lack of the ß-chain interferes with antibody binding

To further explore whether *CLU* β-chain mutations interfere with normal CLU processing in isogenic cell lines, cleaved secreted CLU (sCLU) and uncleaved full-length CLU (FL-CLU) levels were measured in HEK Flp-In cells stably transformed with plasmids encoding CLU wild-type and mutations (Fig. 4). On western blot, overexpression of CLU p.R338W and p.I360N resulted in decreased sCLU levels in cell medium (CM) and increased amounts of FL CLU in cellular lysate (CL) relative to wt CLU (Fig. 4a). ELISA measurements on HEK Flp-In cells showed that for wt cells, 91 % of total CLU was secreted by the cells while 9 % of total CLU was present in CL. Compared to this ratio in wt cells, reduced ratios and sCLU levels were detected for p.R338W cells (68/32 ratio (95 % C.I. 0.11-0.28); *p* < 1×10^−4^) and p.I360N cells (15/85 ratio (95 % C.I. 0.01-0.03); *p* < 1×10^−4^), indicating CLU is retained intracellularly (Fig. 4b). These results were in line with quantification of the western blot bands (Fig. 4c). Identical findings were observed in HEK293T cells where p.R338W and p.I360N showed reduced sCLU in CM versus FL-CLU in CL (p.R338W: 52.8/47.2 ratio (95 % C.I. 0.15-0.31), *p* < 1×10^−5^; p.I360N: 12.3/87.7 ratio (95 % C.I. 0.02-0.05), *p* < 1×10^−5^) compared to wt (76.6/23.4 ratio) (Additional file 4: Figure S4).

Since antibodies against the β-chain were used, no protein levels could be measured for HEK-Flp In and HEK293T cells transduced with p.I303NfsX13 lacking the β-chain. None of the other mutations showed the pattern of intracellular CLU retention observed for p.R338W and p.I360N. Of note, however, the sCLU isoform of p.T345M consistently showed lower molecular weight (~30 kDa) (Fig. 4a, Additional file 4: Figure S4a).

## Discussion

The *CLU* gene has been identified as an important novel risk locus for Alzheimer’s disease [1, 2, 14]. After *BIN1* and *PICALM,* the genome-wide significance level of *CLU* (overall p = 2.8×10^−25^) ranks third on the list of the largest AD meta-analysis combining genetic information of 74,046 individuals [14]. As an extracellular chaperone, clusterin interacts with Aβ_1–40_ oligomers and is able to influence both its aggregation and disaggregation by sequestration in AD [15].

We previously hypothesized that not only common *CLU* variants, but rare non-synonymous and small insertion/deletion mutations play a role in AD [3]. The discovery of multiple patient related *CLU* mutations in Belgian, French and Canadian individuals and a significant meta-analysis for rare variants in the β-chain were in favor of this hypothesis. More specifically, we found that rare coding mutations (missense and small insertion-deletions) in the CLU β-chain were associated with AD risk.

Here we explored the possible effect of these *CLU* mutations, including a novel mutation in the β-chain, on subcellular localization and secretion. As observed in wild-type cells, CLU is a secreted protein that is localized to Golgi and to lesser extent to endoplasmic reticulum, both compartments of the classical secretory pathway. Of interest, our findings suggest that Alzheimer-related mutations located at the cystein-rich region of the CLU β-chain (i.e. p.R338W and p.I360N) deregulate normal CLU secretion and lead to protein retention and degradation in ER before subsequent trafficking towards Golgi. This was shown by the increased CLU intensity in ER and concomitant decreased Golgi signal. The same holds true for p.I303NfsX13 for which almost complete ER retention was observed, an expected finding since this variant was predicted to result in a truncated protein. Compared to wild-type cells, the ratio between CLU secretion in cell medium and uncleaved full-length CLU in cell lysate was significantly altered for mutations p.R338W and p.I360N. Of six mutation carriers for whom serum CLU levels could be determined, one patient carrying p.R338W and the p.I360N carrier showed decreased CLU levels in serum compared to average serum CLU in AD patients without *CLU* mutations (*n* = 314) as well as control individuals (*n* = 349). Their levels were within or just below the interquartile range of values observed in the full AD cohort (Additional file 5: Figure S5). Because of small numbers and impossibility to adequately control for stressors that may induce sCLU in vivo these observations should be interpreted with caution.

The pattern of ER retention was observed for 3 out of 10 mutations. The other 7 mutations include two variants observed in both patients and control individuals (p.P322L and p.N369H), a predicted benign variant (p.A309T) and a variant predicted only possibly pathogenic by one *in silico* prediction program (p.E431Q). No effects on CLU localization and secretion were observed for the 3-amino acid deletion, and p.T440M and p.T345M despite being predicted pathogenic. Conceivably, these mutations may influence other CLU functions, such as altered binding to amyloid particles, disturbed microglia receptor binding or disturbed binding of other CLU ligands. Both the 3-amino acid deletion and p.T440M are located outside the disulfide bond region, while p.T345M is located in this region. Of note, secreted CLU of the p.T345M mutant consistently had lower molecular weight (~30 kDa) on western blots, likely reflecting the absence of a possible phosphorylation site, but this needs further exploration.

Both our western blot and ELISA data confirmed reduced CLU secretion for p.I303NfsX13, p.R338W and p.I360N through the secretory pathway and suggest a potential mechanism for pathogenicity of these mutations. Altered cellular localization and in turn reduced secretion to the extracellular space are indicative of improper folding or mis-assembly of the clusterin subunits, suggesting that these observed β-chain mutations interfere with the biosynthetic-secretory CLU pathway. In these patients, the reduced CLU secretory levels throughout life may hamper CLU upregulation and elevation which is noted in the process of AD [16].

An extension of our finding that AD risk is increased with *CLU* mutations that decrease CLU secretion is that other factors reducing CLU secretion may increase risk as well. This is in line with the observation that SNP rs11136000 was associated with increased CLU expression, which in turn may act in reducing AD risk [10]. Moreover, higher clusterin concentrations in plasma have been associated with slower rates of brain atrophy in mild cognitive (MCI) patients [17].

The use of a cell over-expression system is unlikely to have biased our findings since consistent secretory localization patterns for intact full length CLU were observed. Furthermore, CLU-p.insA cells express truncated CLU which leads to a distorted localization pattern that is consistent with protein degradation. Therefore our assay was suitable for observing the effect of high impact mutations. While strongest CLU expression is found in brain astrocytes, the stretched phenotype of HeLa cells allows accurate cytoplasm localization, and isogenic Flp-In cells have constant protein expression, making these cell types most suitable in first-line characterization of mutations interfering with CLU localization and secretion. Moreover previous studies have shown that stable transfected HEK293 cells produced similar CLU protein as human plasma protein in terms of glycosylation, proteolytical cleavage into α and β-subunits and chaperone activities [18].

A limitation of the present study is that only the secretory CLU isoform has been investigated, selected because of its predominance in brain and chaperone activities towards Aβ and role in lipid metabolism in AD. A recent study exploring the role of intracellular CLU (iCLU) in AD, reported an interaction between iCLU and tau and BIN1 protein [19]. No interaction was detected between BIN1.1 and iCLU for p.I303NfsX13 (denoted as MT8 in [19]), providing evidence that the CLU-BIN1 interaction occurs through their coiled-coil motifs [19]. Interestingly, an increased generation of iCLU (50 kDa) relative to sCLU (37 kDA) was found for p.R338W (MT1 in [19]), which is in line with our observation of decreased p.R338W secretion relative to full-length CLU *in vitro*. Following transfection of the secreted CLU isoform, we did not observe the intracellular CLU isoform but the finding that full length CLU is elevated for p.R338W and p.I303NfsX13 shows that these mutations are implicated in disease etiology. Mutations that diminish CLU secretion may potentially modify AD risk by hampering CLU upregulation and protective mechanisms during stress and inflammation (e.g. amyloid clearance). Alternatively, the combination of reduced CLU protection and increased CLU cytotoxicity may be involved in lowering the threshold of disease onset.

## Conclusions

Identification of rare mutations in Alzheimer risk genes may provide additional insights in the molecular mechanisms underlying a genetic association. Moreover, the ability to functionally characterize the effect of rare variants will become increasingly pressing in the era of massive parallel re-sequencing. The data presented here lend further support to the contribution of rare coding *CLU* mutations in the pathogenesis of Alzheimer’s disease, and suggest reduction of secreted CLU as a possible disease mechanism, specifically for a frameshift mutation and two mutations altering the disulfide bridge region of the protein. The mode of action of the other predicted pathogenic mutations warrants further investigation.

## Methods

### Population descriptives

Since publication of our previous data [3], 74 additional Alzheimer patients (mean age of onset 82 ± 11.3 years, %female 56.3) were screened for *CLU* coding exons. Patients were ascertained at the Memory Clinic of the ZNA Middelheim and Hoge Beuken, Antwerp, Belgium (S.E.,P.P.D.D.) in the frame of a prospective study of neurodegenerative and vascular dementia in Flanders, the Dutch-speaking region of Belgium [20, 21], and at the Memory Clinic of the University Hospitals of Leuven (UHL), Gasthuisberg, Leuven, Belgium (M.V.,R.V.) as part of a prospective study on the molecular genetics of cognitive impairment using the same clinical assessments and biosampling schemes. Each patient underwent a neuropsychological examination and structural and/or functional neuroimaging [22].

### Genetic screening

We designed a PCR-based target enrichment assay using MASTR™ technology (Multiplicom, Niel, Belgium) including fourteen *CLU* fragments (3.3 kb) covering the 9 *CLU* coding exons, flanking intron-exon boundaries and UTR regions (NM_001831.2 and NM_20339.1). Primers for multiplex PCR were designed using mPCR. Multiplex PCR was performed for amplification of the target region, followed by purification of the pooled amplicon library using Agencourt AMPureXP beads (Beckman Coulter, CA, USA). Patient-specific barcodes (Multiplicom, Niel, Belgium) were incorporated during a universal PCR step. Barcoded samples were pooled prior to bridge amplification and sequencing on MiSeq, using the reagent kit v2, generating 250 bp paired-end reads (Illumina, San Diego, CA, USA). Data was processed with the Illumina pipeline and Genomecomb’s [23] data annotation and filtering package, including read alignment against build GRCh37/hg19 and mapping steps with Burrows-Wheeler Aligner (BWA) [24] and variant calling with Genome Analysis Toolkit (GATK) [25]. Rare *CLU* variants were filtered on impact (frameshift, nonsense, non-synonymous and splice variants), low-frequency in 1000 Genomes Project [26] (frequency <0.01) and European American samples of the NHLBI GO Exome Sequencing Project (ESP6500 data set) (frequency <0.01). Filtered variants were visually inspected using interactive genomics viewer (IGV). In total, 78.5 % of the target region had a coverage ≥ 50x in all samples. Rare *CLU* variants were validated using Sanger sequencing as described before [3]. The effects of non-synonymous substitutions on protein function were evaluated with PolyPhen2 [5], Pmut [27] and SIFT [4] programs.

### cDNA constructs

The effect of *CLU* mutations on CLU secretion was modeled using a synthetic cDNA construct of NM001831.3 (GeneCopoeia), starting from the second ATG codon for exclusive expression of the short secreted isoform (449 AA) [10]. Using attB sites containing primers, CLU ORF was amplified and subsequently recombined into the pDONR221 vector using the Gateway Cloning System (Life Technologies). In total, 11 *CLU* missense and small insertion-deletion mutations were introduced in this pDONR construct by site-directed mutagenesis using Kapa High Fidelity Polymerase. All sequences were verified using Sanger sequencing and subsequently transferred to pEGFPC-GW, an in-house designed gateway-compatible destination vector with C-terminal EGFP tag (without stopcodon), to allow the expression of CLU-EGFP fusion proteins.

In addition, the ORFs from wild-type and CLU mutants (with stop codon) were cloned into the pEF/FRT/V5 Gateway vector (Life Technologies) and the pLENTI6/V5 vector (Life Technologies) to generate stable CLU expressing cell lines by Flp recombinase-mediated integration, or by lentiviral vector transduction, respectively.

### Cell culture

HeLa cells (Henrietta Lack cells, cervical cancer cells; ATCC) were maintained in Minimal Essential Medium (MEM) supplemented with 10 % (v/v) fetal bovine serum, 100 U/ml penicillin/streptomycin at 37 °C in a humidified 5 % CO_2_ atmosphere.

Flp-In HEK293 cells (Life technologies), stable Flp-In cell lines and transduced HEK293T cells expressing the *CLU* gene were cultured in Dulbecco’s modified Eagle’s medium (DMEM), supplemented with Zeocin (400 μg/ml, Life Technologies, HygromycinB (200 μg/ml, Ducheva Biochemie) or Blasticidin (4 μg/ml, Invivogen) respectively.

### Lentiviral vector production

Human immunodeficiency virus type 1 (HIV-1)-derived lentiviral vectors were produced by a standardized three plasmid transient transfection protocol [28]. Briefly, HEK293T cells were transfected with a second-generation packaging plasmid pCMV dR8.91, an envelope plasmid encoding the VSV-G protein, and the pLenti6/V5 transfer plasmid carrying the *CLU* CDS with mutations of interest using calcium phosphate as transfection reagent. The cell supernatant containing the lentiviral particles was harvested 48 h post-transfection, filtered using a 0.45 μm PVDF membrane (Millex), aliquoted and stored at −20 °C. The total amount of LV was determined by measuring the lentivirus associated p24 antigen content using a commercial enzyme linked immunosorbent assay (HIV-1 p24 ELISA, Cell Biolabs).

### Transfections and lentiviral transductions

Nearly confluent HeLa cells were transfected in 35 mm dishes with plasmids encoding the wild-type and mutant CLU-EGFP fusion protein using Lipofectamine 2000 (Life Technologies) according to the manufacturer’s recommendations.

Secondly, to generate stable CLU overexpressing HEK Flp-In cell lines, 1.2×10^6^ cells per 100 mm plate were co-transfected with a total of 10 μg DNA of the pEF/FRT/V5 and pOG44 plasmids at a 1/9 ratio, using Lipofectamine 2000 (Life technologies). Cells were selected with HygromycinB antibiotic after 48 h. Integration of the *CLU* wild-type and mutant cDNA was sequence-verified on gDNA isolated from these cells (DNA blood minikit, Qiagen).

Thirdly, HEK293T cells (HCL4517, Fermentas) were transduced for 24 h with various LV vectors normalized to P24 antigen (infectious titer of 2.10^7^ TU). Selection in DMEM media containing Blasticidin resulted in stable polyclonal CLU expressing HEK293T cell lines.

### Live cell imaging and immunocytochemistry on HeLa cells

Examination of living (unfixed) HeLa cells was performed 48 h after transfection with CLU-EGFP expressing constructs. Cells grown on glass bottom imaging dishes (MatTek Corporation) were imaged on a Zeiss LSM 700 confocal microscope equipped with a stage top incubator (Pecon Gmbh), maintaining conditions of 37 °C and 5 % CO_2_.

For immunocytochemical staining, HeLa cells grown on glass coverslips were fixed in 3 % paraformaldehyde (PFA) at 48 h after transfection with the CLU-EGFP constructs. After washing with PBS and blocking for 1 h with Donkey Serum (1:500, Abcam, Cambridge) at 37 °C in PBT (PBS + 0.02 % Triton-X-100, 0.5 % BSA), cells were incubated for 1 h at room temperature with goat anti-EGFP antibody (1:2000, Abcam, Cambridge) together with a marker for either Golgi (rabbit anti-giantin, 1:1000, Covance, Rotterdam, The Netherlands) or ER (mouse anti-PDI, 1:100, Abcam, Cambridge). Subsequently, secondary antibody incubation for 1 h at room temperature (Donkey anti-Rabbit Alexa Fluor 594 and Donkey anti-Goat, both at 1:500, Life Technologies), F-actin cytoskeleton staining (Phalloidin conjugated to Alexa Fluor 647 (25 min, 1:30, Life Technologies) and nuclear DAPI staining (4’, 6-diamidino-2-phenylindol, 100 ng/ml, 10 min, Bio-Rad, Nazareth Eke, Belgium) was performed with intermittent washes in PBT. After a final wash step with PBS, sections were mounted using DAKO fluorescent mounting medium and stored at 4 °C in the dark before being subjected to fluorescence microscopy.

### Image acquisition and quantification

Confocal images used for quantification (1892×1892, 106×106 nm^2^ pixels) were acquired on a Zeiss LSM 700 microscope with Zen 2009 software (Zeiss, Zaventem, Belgium), using a EC Plan-Neofluar 40x /1.30 Oil objective, while keeping identical acquisition settings (laser intensities, detector gain, channel settings). Images of EGFP-CLU and the organelle markers (ER or Golgi) were acquired in different tracks (serial frame scanning) to avoid any possible crosstalk between the channels. Images were acquired and quantified with genotype labels blinded for the investigators.

ImageJ [29] was used to measure both the relative EGFP intensity in the Golgi and the colocalization with the ER. In both cases the F-actin cytoskeleton staining was first used to manually delineate the regions of the cells, which were stored in ImageJ ROI manager files. For quantification of the relative EGFP signal in the Golgi, the Golgi region was segmented by using an automatic threshold followed by filtering of the binary mask. The ratio of mean EGFP intensity in and outside the Golgi was calculated, corrected for the background signal, and based upon the mean intensity in the Golgi and the mean intensity outside Golgi in the remaining cell region. A fixed cutoff threshold of EGFP mean intensity outside the Golgi region was used to exclude cells with low EGFP signal. A minimum of 20 cells per genotype were used to perform quantification. To quantify EGFP colocalization with PDI, the ImageJ Coloc2 plugin was used to measure the Pearson’s correlation coefficient between the two fluorescent channels in individual cells. For both types of measurements (ER and Golgi), ImageJ scripts (macro) were written and used to process and analyze all images (and all genotypes) in batch.

### Protein extraction

Both HEK Flp-In and HEK293T cells were grown to confluence in a 100 mm dish format. After washing with Dulbecco’s Phosphate-buffered saline (PBS), cell medium was replaced by serum-free OPTIMEM I medium (Life Technologies). Cell media and cell lysates were harvested 24 h later. One ml of cell medium, supplemented with the protease inhibitor AEBSF (1 mM final, Calbiochem), was centrifugated (15.000 g for 5 min, 4 °C). Supernatants were aliquoted and immediately stored at −80 °C. After an initial wash with PBS, cell lysates were prepared by collecting the cells with a cell scraper followed by lysis with 2 ml NP40 buffer (Life Technologies) supplemented with the protease inhibitor AEBSF. The cell lysates were incubated on ice for 10 min, centrifugated (15.000 g for 5 min, 4 °C) and immediately stored at −80 °C.

### CLU ELISA

Secreted CLU from conditioned medium and CLU from cell lysates of HEK293T cells was quantified by a commercial CLU ELISA (Human Clusterin Elisa RD194034200R, Biovendor, Germany). All undiluted samples were run in duplicate. Besides the quality control samples provided by the manufacturer, inter-assay samples were run in each experiment. Three independent experiments were performed per mutation. Normalization was performed using TGF-ß1. Because of a slight immunoreactive response of the sample matrix, as observed in measurement of nontransfected cells, the average of the immunoreactive response was computed across three experiments and subtracted from the observed data for each mutation.

### Western blot and immunodetection

Protein concentrations were determined with a bicinchoninic acid colorimetric assay (BCA) (ThermoScientific). Equal amounts of protein (20 μg) were loaded and separated on NuPAGE® 10 % Bis-Tris gels (Life Technologies). Proteins were transferred onto polyvinylidene difluoride membranes (Hybond P, Amersham Biosciences). After blotting, membranes were blocked in 5 % skimmed milk in PBS containing 0.1 % Tween® 20 (Merck) (PBT) for 2 h at room temperature and probed overnight with a mouse anti-clusterin antibody (1:200, B-5 Santa Cruz recognizing the CLU β–chain) at 4 °C. Equal loading was controlled using a mouse α-tubulin antibody (1:10000, GeneTex). Immunodetection was achieved with horseradish peroxidase (HRP)-conjugated sheep anti-mouse antibody (1:10000, BioSciences) and an ECL Plus™ chemiluminescent detection system (GE Healthcare). Bands were visualized and quantified using ImageQuant LAS 4000 equipment (GE Healthcare Life Sciences).

### Statistical analysis

For quantification of the colocalization experiments, Pearson’s R correlation value was determined between ER and CLU reactivity (using ImageJ Coloc2 plugin). For Golgi measurements, ratios of mean intensity inside Golgi versus outside Golgi apparatus were determined. Means of the independent measurements were compared to wt using ANOVA (Tukey HSD test). Extreme outliers (*n* = 23 from 568 measurements), defined as values with more than 3 standard deviations from the mean were removed from the analysis.

For quantification of western blot data, CLU protein levels in cell media and lysates were evaluated through quantification of band intensities with ImageQuant software (GE Healthcare Life Sciences). Independent measures of CLU intensity ratios between cell lysates and cell media were used for further analysis. Cochran-Mantel-Haenszel test was used to compare ratios of CLU in cell medium versus lysate between CLU wt and mutant genotypes. Bonferroni correction for 11 different constructs is applied. P-values <0.0045 are considered significant. Results are represented as mean ratios of CLU in cell medium and lysate (Fig. 4, Additional file 4: Figure S4).

### Consent

All participants and/or legal guardians gave written informed consent for participation in genetic studies, and for publication of study results, in an anonymized manner. Clinical study protocol and informed consent forms for ascertainment were approved by the Ethics Committee of the respective hospitals at the sampling sites. Genetic study protocols and informed consent forms were approved by the Ethics Committees of the University of Antwerp and the University Hospital of Antwerp, Belgium.

## Additional files

Additional file 1: Figure S1. The distribution of CLU-EGFP wt (top) and CLU mt p.I360N (bottom) in Golgi and ER. CLU localization is shown for the entire cell and for enlarged regions in the perinuclear region and the cell periphery of the same cell. For wild-type CLU the most intense EGFP signal was present in the Golgi, and in vesicles in the cytoplasm in addition to the ER. For p.I360N, CLU EGFP seemed almost exclusively present in the ER. No CLU-EGFP was present in the nucleus. Additional file 2: Figure S2. The distribution of CLU-EGFP wt and CLU mutations in relation to the ER. Localization of CLU (EGFP) and the ER (PDI marker) is shown for a 21 μm × 21 μm region containing the perinuclear zone of cells expressing the different CLU genotypes. For p.I303NfsX13, p.R338W and p.I360N CLU EGFP is almost exclusively present in ER, while for wild-type and the other mutations the most intense signal is present in Golgi-resembling structures. Scale bar = 5 μm. Additional file 3: Figure S3. The distribution of CLU-EGFP wt and CLU mutations in relation to the Golgi. Localization of CLU (EGFP) and the Golgi (Giantin marker) is shown for a 21 μm × 21 μm region containing the perinuclear zone of cells expressing the different CLU genotypes. The most intense CLU signal is present in Golgi, except for p.I303NfsX13, p.R338W and p.I360N, which contain little CLU in the Golgi. Scale bar = 5 μm. Additional file 4: Figure S4. CLU secretion is altered for *CLU* coding variants in HEK293T cells. (a) Variants p.R338W and p.I360N showed reduced expression in CM and increased expression in CL in HEK293T cells. Α-tubulin staining is incorporated as an equal loading control (b) Immunoblot quantification of the CLU ratio of CM/CL for different CLU overexpressing HEK293T cell lines revealed alterations for p.R338W and p.I360N compared to wt CLU (***p* < 1×10^−5^). Additional file 5: Figure S5. Serum CLU levels of CLU mutation carriers. Log_10_-transformed circulating levels of CLU in serum of six mutation carriers determined by ELISA. For p.R338W and p.T445_D445del, serum CLU levels were determined in two carriers. For these mutations, the diamond indicates the average of two carriers, and caps on bars indicate the individual levels per carrier. The blue dashed line represents the mean log transformed CLU serum concentration in 314 AD patients (1.93 μg/ml). The blue dotted lines indicate lower and upper boundary of the 95 % confidence interval (CI) of the mean (1.913 – 1.949 μg/ml). The red dotted lines indicate the interquartile range (IQR 1.822 – 2.053 μg/ml). Values for control individuals (*n* = 349; not shown in the figure): mean 1.931 μg/ml, 95 % CI 1.914 – 1.947 μg/ml), IQR 1.821 – 2.036 μg/ml).

## Abbreviations

AD: Alzheimer’s disease
CL: Cell lysate
CLU: Clusterin
CM: Cell medium
FL-CLU: Full-length CLU
ER: Endoplasmic reticulum
EVS: Exome Variant Server
iCLU: Intracellular CLU
MASTR: Multiplex amplification of specific targets for resequencing
mt: Mutant
sCLU: Secreted CLU
SIFT: Sorting intolerant from tolerant
SNP: Single nucleotide polymorphism
1000GP: 1000Genomes Project
wt: Wild-type
