# Supplementary figures and images for: Reduced secreted clusterin as a mechanism for Alzheimer-associated CLU mutations
Source: Mol Neurodegener. 2015 Jul 16;10:30. doi: 10.1186/s13024-015-0024-9 (PMC4502563; doi:10.1186/s13024-015-0024-9)

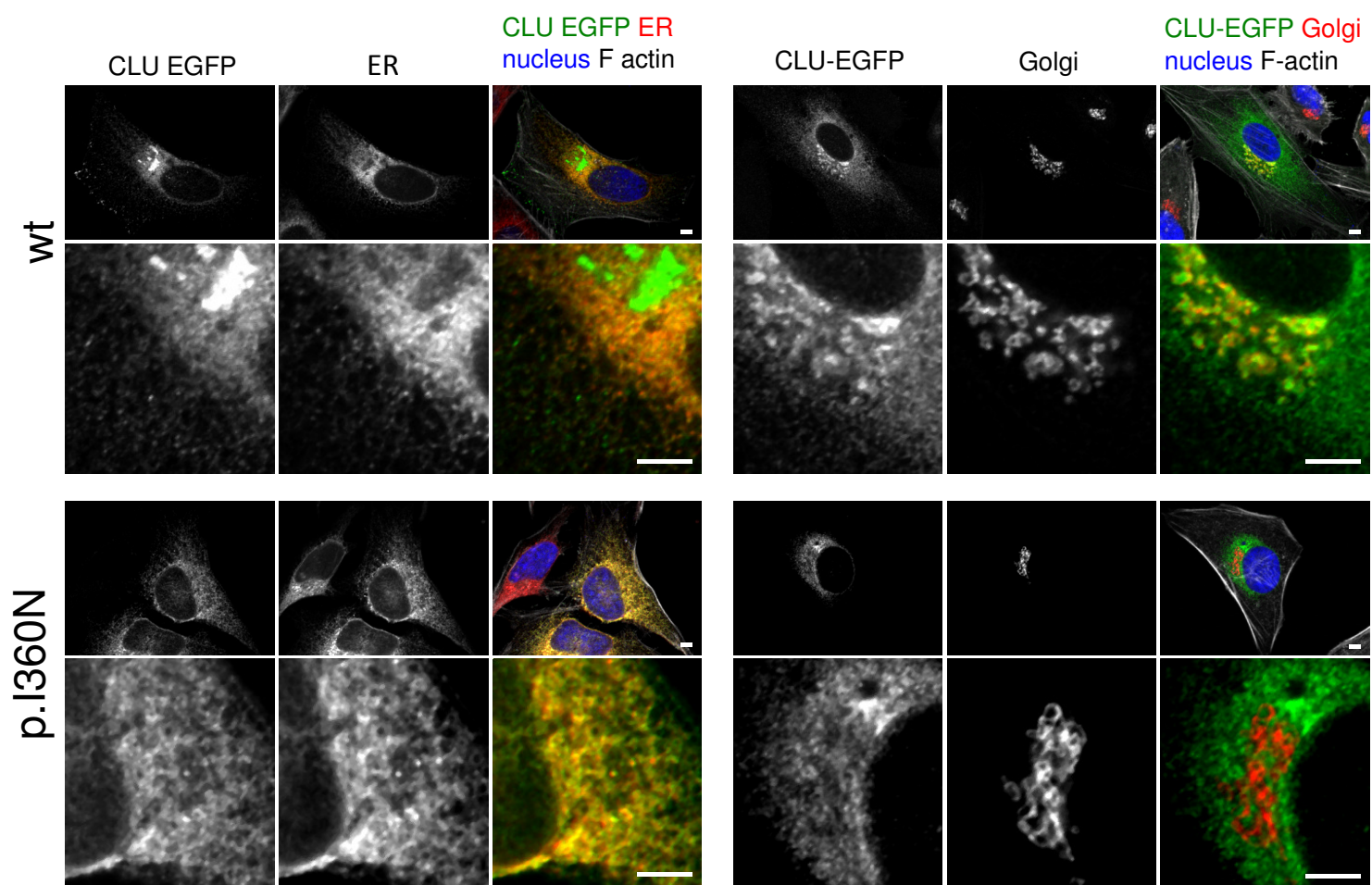

Supplement: Additional file 1: Figure S1. — The distribution of CLU-EGFP wt (top) and CLU mt p.I360N (bottom) in Golgi and ER. CLU localization is shown for the entire cell and for enlarged regions in the perinuclear region and the cell periphery of the same cell. For wild-type CLU the most intense EGFP signal was present in the Golgi, and in vesicles in the cytoplasm in addition to the ER. For p.I360N, CLU EGFP seemed almost exclusively present in the ER. No CLU-EGFP was present in the nucleus. [file 13024_2015_24_MOESM1_ESM.pdf]

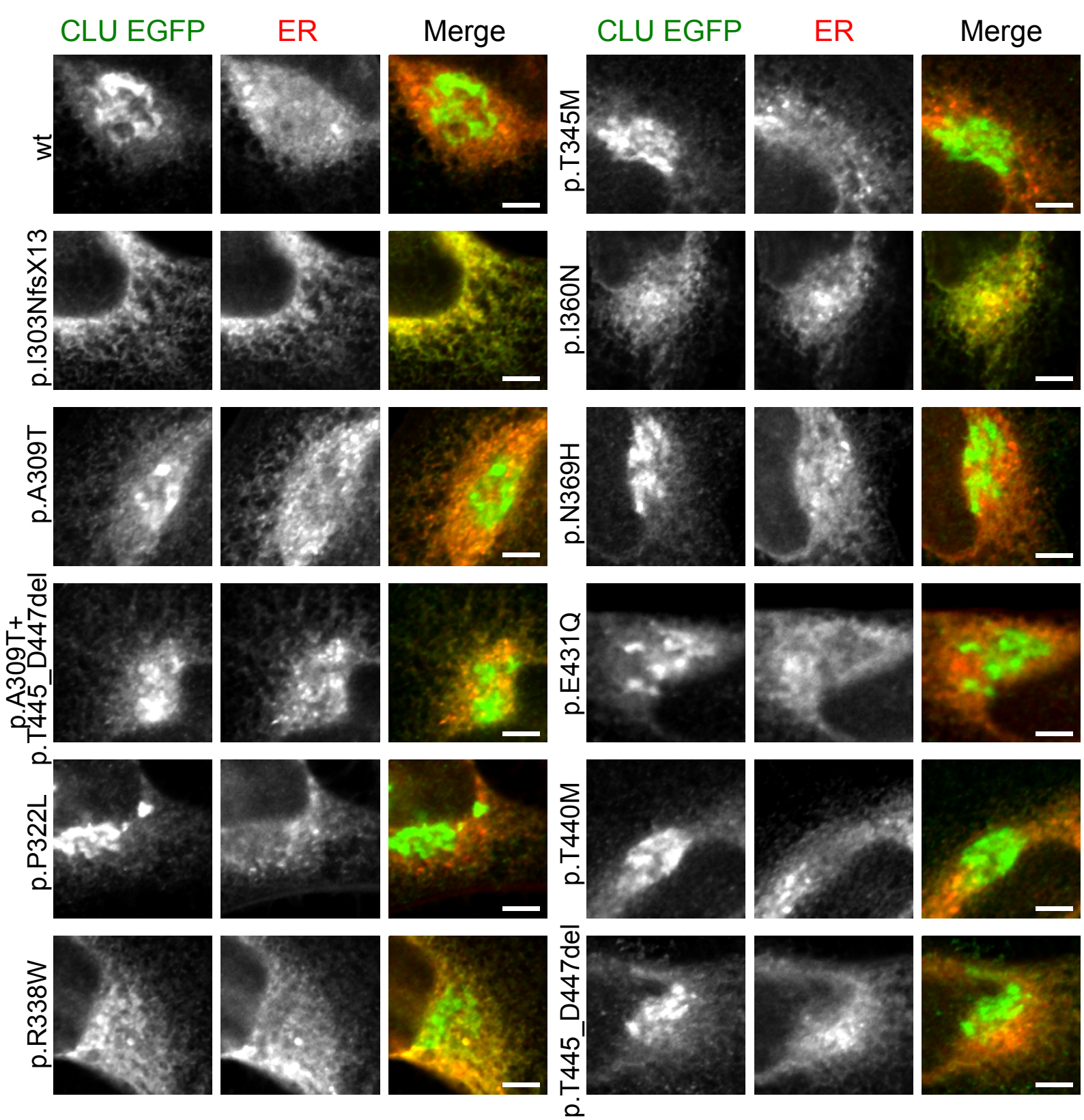

Supplement: Additional file 2: Figure S2. — The distribution of CLU-EGFP wt and CLU mutations in relation to the ER. Localization of CLU (EGFP) and the ER (PDI marker) is shown for a 21 μm × 21 μm region containing the perinuclear zone of cells expressing the different CLU genotypes. For p.I303NfsX13, p.R338W and p.I360N CLU EGFP is almost exclusively present in ER, while for wild-type and the other mutations the most intense signal is present in Golgi-resembling structures. Scale bar = 5 μm. [file 13024_2015_24_MOESM2_ESM.pdf]

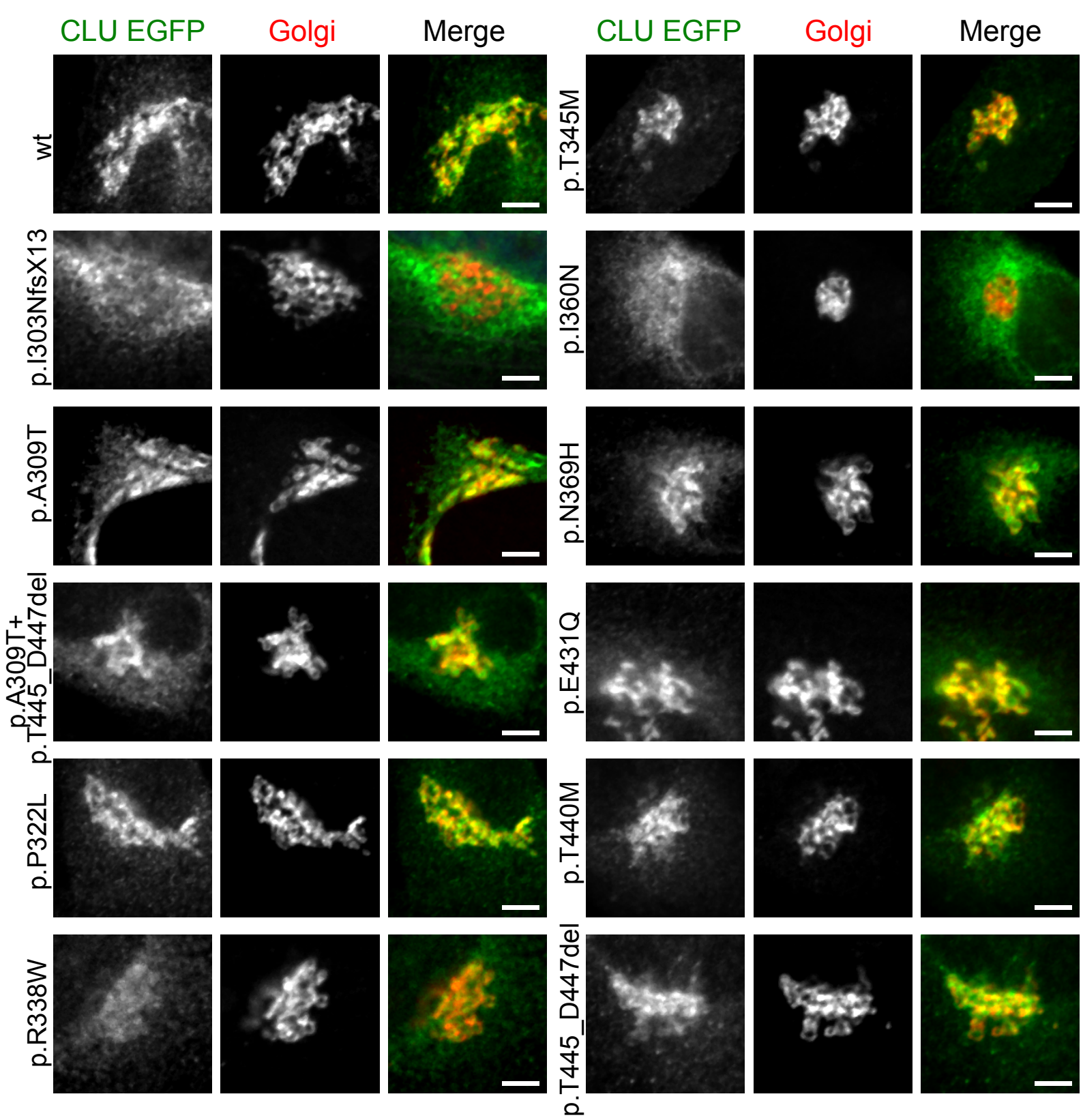

Supplement: Additional file 3: Figure S3. — The distribution of CLU-EGFP wt and CLU mutations in relation to the Golgi. Localization of CLU (EGFP) and the Golgi (Giantin marker) is shown for a 21 μm × 21 μm region containing the perinuclear zone of cells expressing the different CLU genotypes. The most intense CLU signal is present in Golgi, except for p.I303NfsX13, p.R338W and p.I360N, which contain little CLU in the Golgi. Scale bar = 5 μm. [file 13024_2015_24_MOESM3_ESM.pdf]

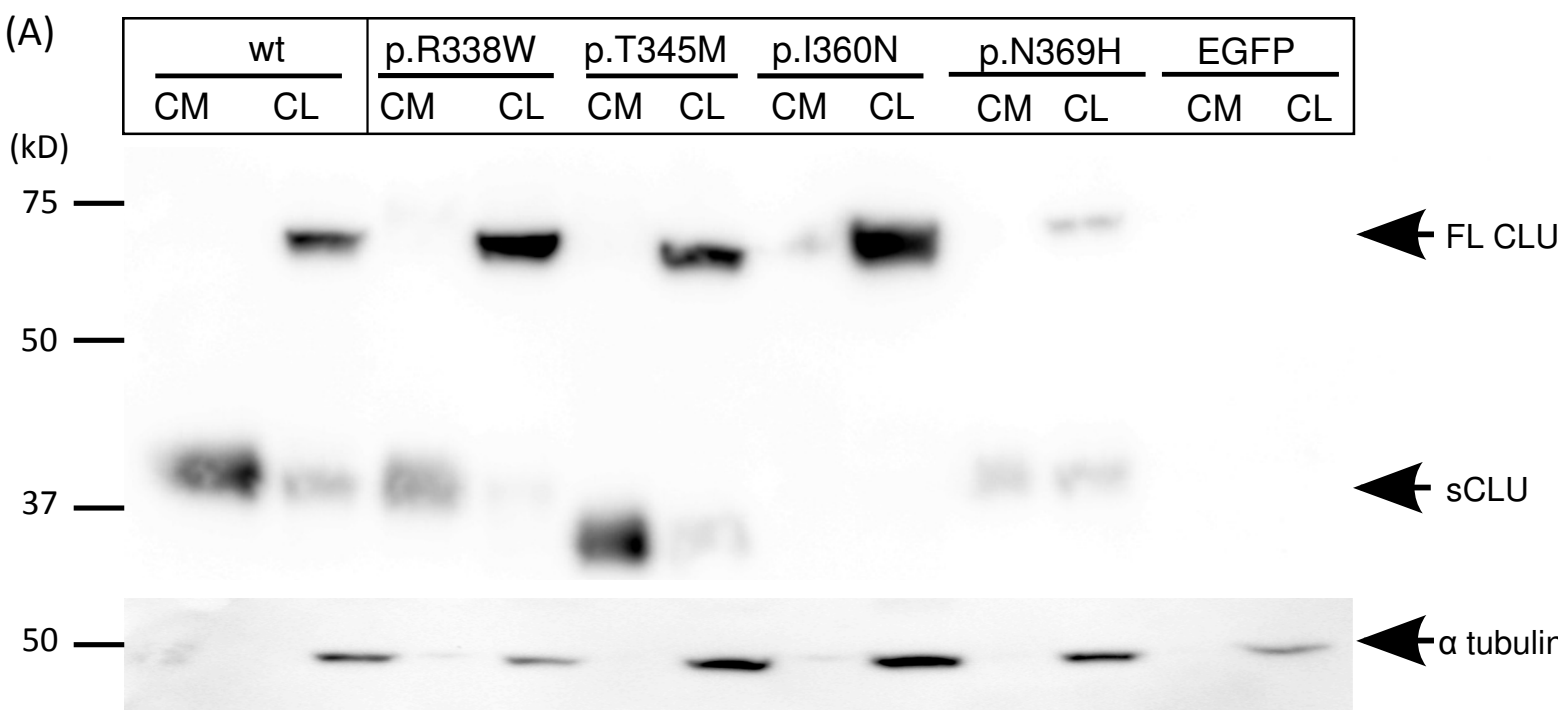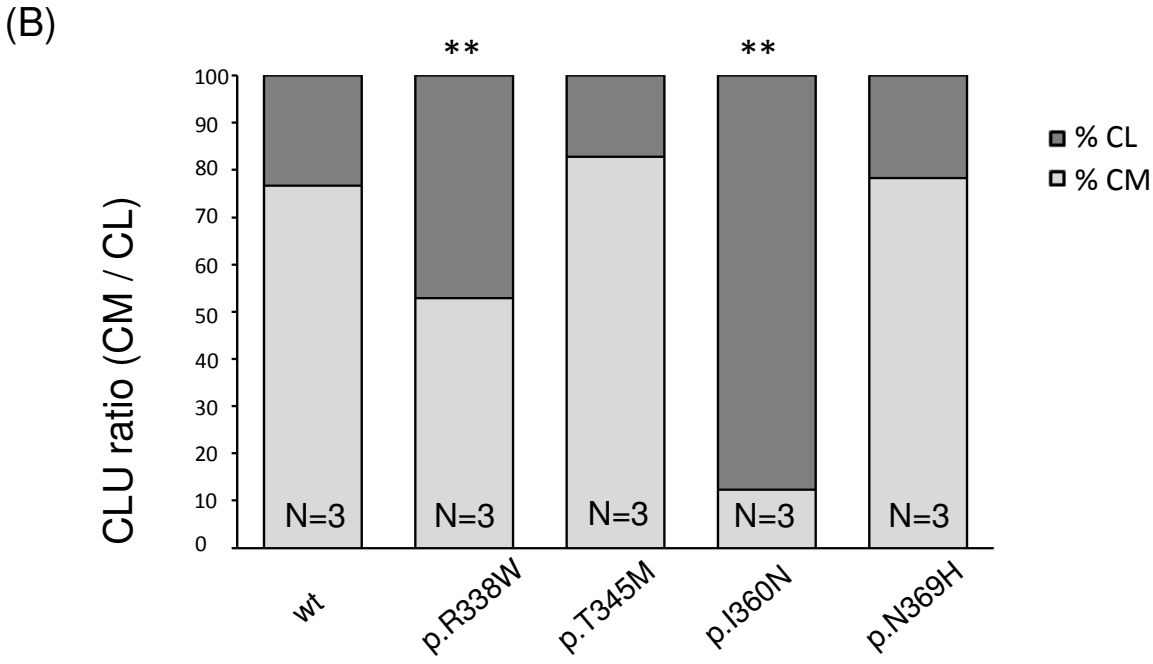

Supplement: Additional file 4: Figure S4. — CLU secretion is altered for CLU coding variants in HEK293T cells. (a) Variants p.R338W and p.I360N showed reduced expression in CM and increased expression in CL in HEK293T cells. Α-tubulin staining is incorporated as an equal loading control (b) Immunoblot quantification of the CLU ratio of CM/CL for different CLU overexpressing HEK293T cell lines revealed alterations for p.R338W and p.I360N compared to wt CLU (**p < 1×10^−5^). [file 13024_2015_24_MOESM4_ESM.pdf]

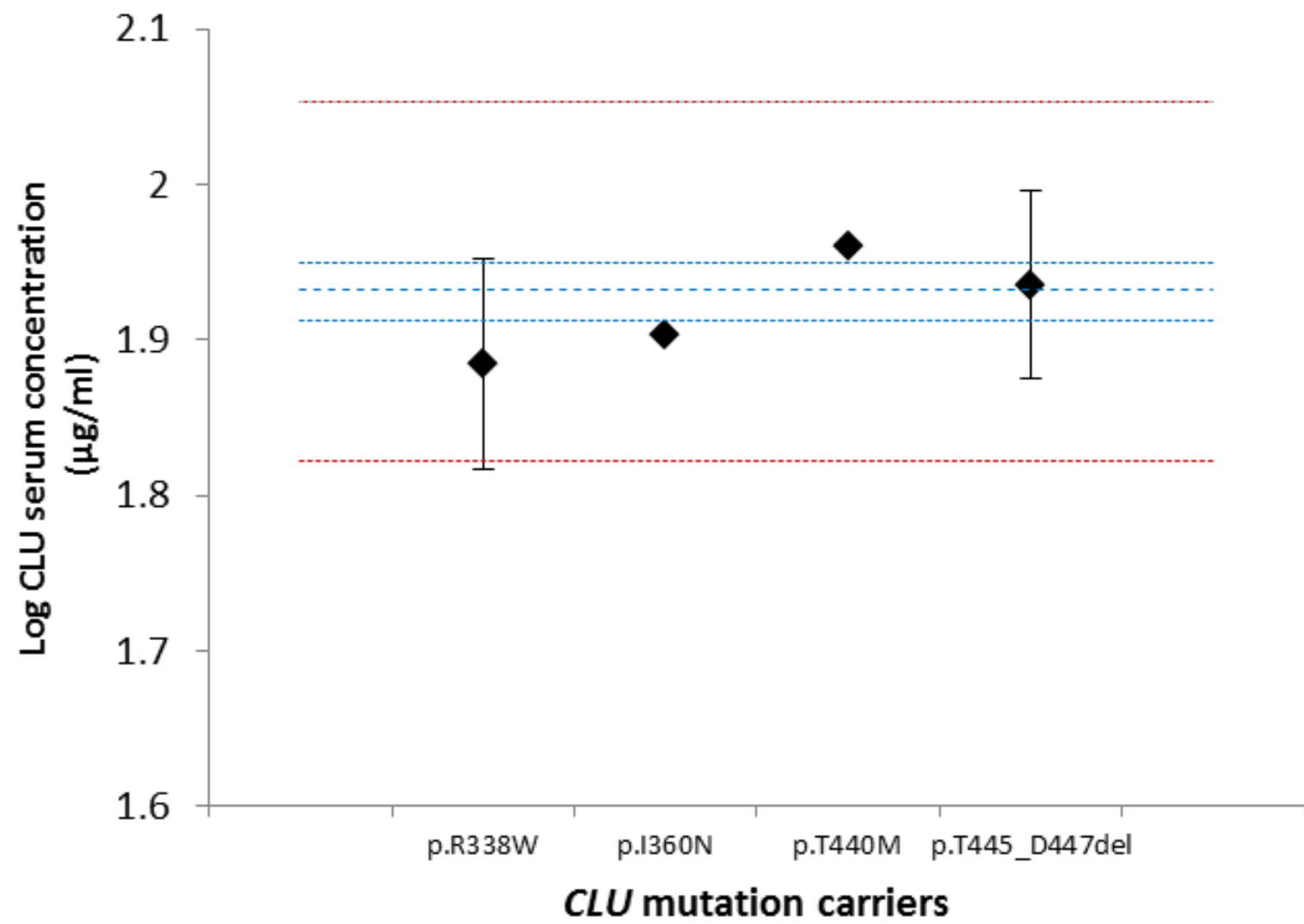

Supplement: Additional file 5: Figure S5. — Serum CLU levels of CLU mutation carriers. Log10-transformed circulating levels of CLU in serum of six mutation carriers determined by ELISA. For p.R338W and p.T445_D445del, serum CLU levels were determined in two carriers. For these mutations, the diamond indicates the average of two carriers, and caps on bars indicate the individual levels per carrier. The blue dashed line represents the mean log transformed CLU serum concentration in 314 AD patients (1.93 μg/ml). The blue dotted lines indicate lower and upper boundary of the 95 % confidence interval (CI) of the mean (1.913 – 1.949 μg/ml). The red dotted lines indicate the interquartile range (IQR 1.822 – 2.053 μg/ml). Values for control individuals (n = 349; not shown in the figure): mean 1.931 μg/ml, 95 % CI 1.914 – 1.947 μg/ml), IQR 1.821 – 2.036 μg/ml). [file 13024_2015_24_MOESM5_ESM.pdf]
